# Pathways to global-change effects on biodiversity: new opportunities for dynamically forecasting demography and species interactions

**DOI:** 10.1098/rspb.2022.1494

**Published:** 2023-02-22

**Authors:** Maria Paniw, David García-Callejas, Francisco Lloret, Ronald D. Bassar, Joseph Travis, Oscar Godoy

**Affiliations:** ^1^ Department of Conservation Biology and Global Change, Estación Biológica de Doñana (EBD-CSIC), Seville, 41001 Spain; ^2^ Department of Integrative Ecology, Estación Biológica de Doñana (EBD-CSIC), Seville, 41001 Spain; ^3^ Department of Evolutionary Biology and Environmental Studies, University of Zurich, Zurich 8057, Switzerland; ^4^ Instituto Universitario de Investigación Marina (INMAR), Departamento de Biología, Universidad de Cádiz, Campus Río San Pedro, 11510 Puerto Real, Spain; ^5^ Center for Ecological Research and Forestry Applications (CREAF), Cerdanyola del Vallès 08193, Spain; ^6^ Department Animal Biology, Plant Biology and Ecology, Universitat Autònoma Barcelona, Cerdanyola del Vallès 08193, Spain; ^7^ Department of Biological Sciences, Auburn University, Auburn, AL 36849, USA; ^8^ Department of Biological Science, Florida State University, Tallahassee, FL 32306, USA

**Keywords:** size-dependent biotic interactions, forecast bias, Bayesian hierarchical models, latent state, integrated ecological forecasts, metapopulations

## Abstract

In structured populations, persistence under environmental change may be particularly threatened when abiotic factors simultaneously negatively affect survival and reproduction of several life cycle stages, as opposed to a single stage. Such effects can then be exacerbated when species interactions generate reciprocal feedbacks between the demographic rates of the different species. Despite the importance of such demographic feedbacks, forecasts that account for them are limited as individual-based data on interacting species are perceived to be essential for such mechanistic forecasting—but are rarely available. Here, we first review the current shortcomings in assessing demographic feedbacks in population and community dynamics. We then present an overview of advances in statistical tools that provide an opportunity to leverage population-level data on abundances of multiple species to infer stage-specific demography. Lastly, we showcase a state-of-the-art Bayesian method to infer and project stage-specific survival and reproduction for several interacting species in a Mediterranean shrub community. This case study shows that climate change threatens populations most strongly by changing the interaction effects of conspecific and heterospecific neighbours on both juvenile and adult survival. Thus, the repurposing of multi-species abundance data for mechanistic forecasting can substantially improve our understanding of emerging threats on biodiversity.

## 1. Demographic determinants of species responses to environmental change

We are living in an era of unprecedented human-driven perturbations affecting all levels of biological organization, from local populations to communities to entire ecosystems. Such perturbations are complex, often consisting of synergistic, nonlinear effects of multiple abiotic and biotic factors [1,2]. When this complexity of human impacts meets complex natural systems, where different interacting species are differently affected by environmental drivers, it becomes imperative to understand key pathways through which environmental change can alter natural communities [3–5]. Understanding these pathways allows us to define more nuanced ecological forecasting, proposing different scenarios under which populations remain viable in the future, when they go locally extinct, or when they invade new habitats [6–8].

**Figure 1.**
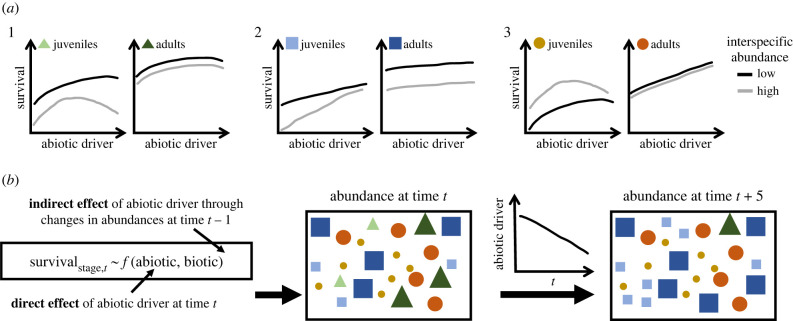
Conceptual overview of how feedbacks between stage-specific demographic rates and species interactions can affect community dynamics under environmental change. As an example (*a*), stage-specific survival rates of three species respond differently to an abiotic driver and biotic interactions (approximated by interspecific abundances): 1—adult survival changes more strongly under abiotic (positive) than biotic (negative) effects; 2—juvenile and adult survival decline more strongly under biotic effects; 3—strong effect of abiotic driver on adult survival, but juvenile survival benefits from high-interspecific abundances. A given value of the abiotic driver at time *t* can therefore (*b*) directly change demographic rates (e.g. survival, recruitment and stage transitions) from time *t* to *t* + 1. This then changes the abundances of species, which, in turn, affect demographic rates and abundances at time *t* + 1, representing an indirect effect of the abiotic driver. Such feedbacks mean that, while all species respond negatively to a decrease in the abiotic driver, unfavourable abiotic conditions may increase the abundance of those species that are more strongly affected by changes in interspecific abundances (e.g. release from competition, blue species here).

In communities that consist of age-, stage- or trait-structured species' populations, a key pathway that needs to be accounted for in robust forecasts are the nuanced effects of global-change drivers across different life cycle stages ([9]; figure 1). For instance, climate-driven changes in the timing of key life cycle events (i.e. phenology) can lead to substantial mismatches in the phenology between species, thus affecting their survival and reproduction [9–12]. However, a lower reproductive output or higher offspring mortality can be compensated by a higher survival of the remaining juveniles and therefore have ultimately little effects on population fitness [13]. These and other cases highlight that global-change drivers do not need to result in changed population dynamics if demographic tradeoff or compensation mechanisms that buffer unfavourable environmental conditions are in place [14–17] or environmental effects are concentrated on demographic rates with low contribution to population growth rates [18]. On the other hand, simultaneous negative environmental effects on several life cycle stages may exacerbate extinction risks [19].

The example of mismatch in the phenology of interacting species demonstrates that the complexity in species’ demographic responses to environmental change is compounded by the complexity of the underlying environmental drivers, where the effects of abiotic drivers on demography can be strongly mediated by biotic interactions (figure 1). That is, species do not exist in isolation in natural communities; both theoretical (e.g. [20,21]) and empirical studies (e.g. [22–24]) on coexistence and trophic interactions show that even relatively small changes in abiotic conditions can alter species interactions and thereby community organization. For instance, changes in competitive interactions can result in niche shifts for some species, thereby allowing for local population persistence even under adverse abiotic conditions [25,26]. Predation pressures have also been shown to constrain adaptive responses to climate change in local populations [27] or to exacerbate adverse climate-change effects [28], while herbivory can be important in maintaining plant community diversity under climate change [29], and the adverse effects of pathogens can be amplified by more extreme climatic events [19]. The effects of species interactions are often specific to particular stages in the life cycles of the interacting species [4,30]. It has been shown that environmental perturbation on key life cycle stages may amplify the effects on negative interactions among species, destabilizing entire ecological communities [31,32]. Such feedbacks between demography and species interactions mean that environmental effects on any populations often scale nonlinearly (or non-additively) to spatiot-emporal abundance changes of neighbouring species (figure 1) [33–35].

Collectively, the examples above suggest that our understanding of changes in biodiversity would be improved if we assess how much feedbacks between demographic rates and species interactions can modify, under increasing abiotic pressures such as climate change, the population persistence of several species in natural communities (figure 1). From a modelling perspective, this means that by parameterizing demographic models as functions of abiotic drivers and species interactions, we can assess how much abiotic drivers affect survival and reproduction directly as opposed to indirectly, when abiotic drivers alter species interactions, e.g. by changing abundances ([36]; figure 1). Only with such information can we understand how lower level demographic effects scale up to affect population dynamics of several species simultaneously and consequently our forecasts of changes in community composition ([33,37,38]; see example in box 1 for interactions between size-dependent demographic responses and intraguild predation).

Box 1. **Individual demographic interactions determine community dynamics.**

One well-studied example that highlights the complexities of species interactions in determining community structure occurs in the high-elevation streams on the Caribbean island of Trinidad. There, Hart's killifish (*Rivulus hartii*) and Trinidadian guppies (*Poecilia reticulata*) compete with each other for resources and consume each other as part of a bidirectional intraguild predation community. Theory predicts that intraguild predation should result in an unstable community, but guppies and killifish coexist in these streams. This is, in part, because the magnitude of interactions between the two species depends strongly on their body sizes (Box Figure).

Simple models of species interactions (e.g. Lotka–Volterra competition equations) describe the negative effects of competition on population growth as simple functions of the numbers, densities, or biomass of competitors in the environment. However, when populations are structured by traits such as body size, simple models like these generate predictions that perform poorly when compared to models that incorporate size-specific interactions between the species [39,40]. For example, for both guppies and killifish, larger individuals are better competitors for a common resource than are smaller ones [41,42]. For guppies, incorporating these differences in projections of their long-term population dynamics produced more accurate results than models assuming all individuals compete equally [30].

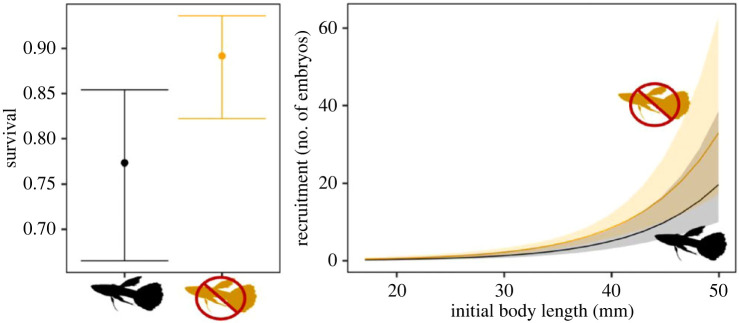

**Box Figure**. Survival and recruitment in killifish vary depending on whether guppies are present (black) or absent (orange) in streams and whether guppies affect all or only certain sizes of killifish. Plot derived from [43].

Yet size often determines more than the competitive ability of the individuals. Larger killifish and guppies also consume resources at higher trophic levels than smaller individuals, which could decrease the negative effects of competition on the smaller fish. As intraguild predators, larger individuals of each species consume smaller individuals of the other species. Because the interactions between these species are so strongly dependent on body size—whether it be competitive interactions or predation—any factor that affects the size distributions of these species can generate markedly different population dynamics and different outcomes of their interaction, coexistence or deterministic competitive exclusion or exclusion of either species via priority effects [40].

Still, the size-based interactions between the two species tell an incomplete story. High-elevation Trinidadian streams contain different microhabitats that each of the species prefers [44]. Recent analyses show that each species may also respond to environmental variation in contrasting ways, with guppies doing well in drier periods and killifish performing better in wetter periods (RDB and JT *unpublished data*). Consider what can happen if climate change was to produce longer dry seasons and drier wet seasons. The pools favoured by guppies may be more productive because of longer periods of high light intensity while the riffles favoured by killifish may shrink in total area from the reduced water flow. As a result, guppies may attain larger body sizes, killifish may grow more slowly and display smaller body sizes, and the size-dependent interactions could result in the elimination of killifish.

Both species also rapidly adapt to the presence of the other species [45,46] and display genetically based morphological, behavioural and life-history differences. These adaptations feedback to alter the rest of the community and ecosystem [47–51]. The outcome of these evolutionary effects is a change in way the species interact over time and for the resulting population dynamics to evolve [43]. These evolutionary changes are repeatable across multiple independent evolutionary instances of guppy/killifish communities, revealing that these evolutionary changes are predictable and hence can, in principle, be incorporated into predictive models. However, this is no small task.

## 2. Shortcomings in forecasting species persistence

Although the intrinsic relationship between demographic and community levels of organization has traditionally been well recognized [52–56], its integration to forecasting has remained elusive [57]. Prominent examples of multi-species demographic models exist in coexistence research [56,58], eco-evolutionary dynamics [59], trophic interactions [60,61] and forest stand dynamics [35]. However, forecasting applications that empirically assess the feedback between species demography and species interactions in a community context are largely missing (but see [35] for approaches to indirectly link demography and community composition via resources). Forecasts that scale from demographic rates to populations focus strongly on a single species and simplify or omit interspecific interactions [62]. This is not surprising as such forecasts rely almost exclusively on long-term individual-based data. This is undoubtedly the most robust approach to quantify demographic processes across the life cycle and link them to emergent population properties [63–65]. However, such datasets are rarely collected for several interacting species. This limitation contrasts with ample evidence showing the role of such interactions in mediating population fates under global-change drivers [31,66–68]. Therefore, a large knowledge gap remains in our understanding of the pathways through which global-change drivers affect the local persistence of multiple interacting species within ecological communities.

## 3. Repurposing abundance data to forecasts feedbacks between demography and species interactions

An important step towards expanding the application of forecasts that integrate feedbacks between demography and species interactions is by repurposing existing long-term abundance datasets. While individual-based data on multiple species in a community are rare, population-level data on stage-specific multi-species abundances are routinely collected in studies focused on spatio-temporal changes in community composition [52,69–73]. Such studies assess relative abundances of species [74–77] or changes in reproduction and survival at the species level [78–80]. However, stage-specific demographic rates are mostly omitted, and studies typically do not explicitly forecast dynamic changes in species interactions. At the same time, forecasting feedbacks between demography and species interactions using multi-species abundance data may have advantages over using individual-based data, especially for applied management. This is because model outputs can be evaluated against population-level data across broad spatial scales [81], which is rarely done with population-level outputs from demographic models based on individual-based data, creating a mismatch in scale [82].

**Figure 2.**
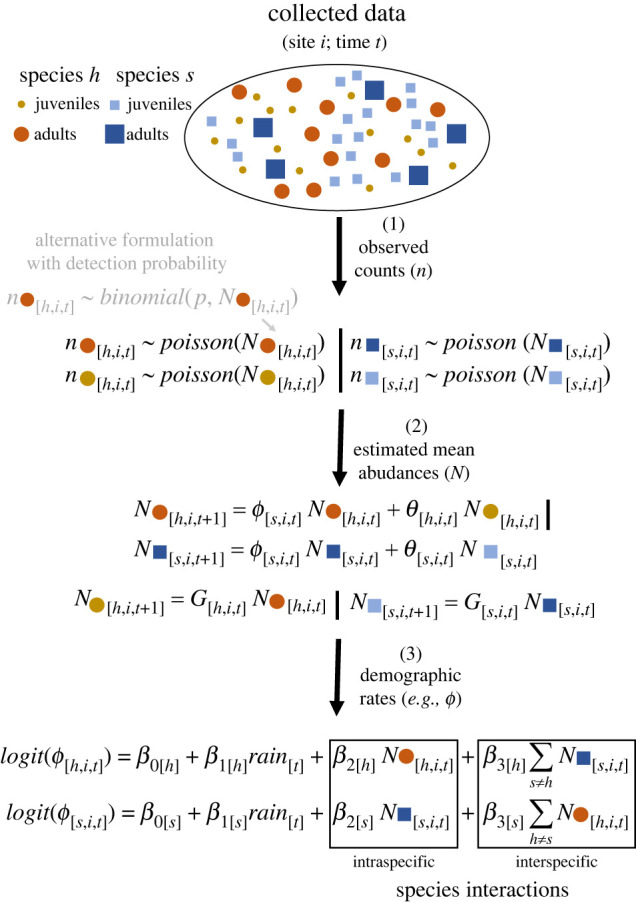
Overview of dynamic N-mixture models to infer demographic rates from stage-specific count data. Using count data (*n*), stage-specific abundances (*N*) for different species (here species *h* and *s*) can be modelled as a function of underlying survival (*θ*—juvenile survival and *φ*—adult survival) and recruitment or gains (G). Under imperfect detection, detection probabilities (*р*) can also be accounted for (shown for species *h*). Species' population dynamics can then be dynamically linked by including the effects of intra- and interspecific abundances in demographic rate models at time *t*, which, in turn, affect abundances at *t* + 1. Here, interspecific effects are represented as the sum of adult abundances for all neighbour species (simplified to one neighbour for clarity) of a focal species. Species can then be jointly projected under a common abiotic driver of demographic rates (e.g. rain).

Recent methodological advances, often termed dynamic N-mixture models, have allowed us to empirically infer the demographic processes underlying species co-occurrences or multi-species abundances (reviewed in [83], see also [84]). Generally, such methods use Bayesian latent-state approaches to estimate gains (recruitment and immigration) and apparent survival (true survival minus emigration) as unobserved processes [85–87] from time series of counts, where such demographic rates can be structured by size, age or stage [84,88], and modelled as a function of density dependence ([89]; figure 2). These approaches offer great potential to link demography and species interactions for multiple species [90–92]. Below, we first discuss some simplifying assumptions that need to be made to parameterize multi-species dynamics forecasts. We then illustrate with an example how to effectively estimate the demographic mechanisms of changes in abundances of several species in a community; we do not consider recent related advances in species range dynamics, which have been discussed in much detail elsewhere [93–96].

## 4. Modelling species interactions in structured demographic models

Inferring age- or stage-specific demographic rates from count data using latent-variable approaches has become increasingly more accessible [83,97]. However, parameterizing such models for interacting species comes with some challenges. The main challenge is the same as those studies inferring species interactions for highly diverse systems [98], namely, how to circumvent the estimation of a large number of parameters [65], some of which may not be identifiable [99]. For instance, if we consider three interacting species (e.g. two herbivores which share a top predator) and three life stages (juvenile, non-reproductive adult and reproductive adult), we need to estimate, at a minimum, 3 × 3 = 9 interaction effects, typically modelled as densities of con- and interspecific neighbours [56,99], for each demographic stage. This equals to 27 pairwise interactions determining the survival, development and reproduction of the three species. We would also need to add additional parameters that map the effect of environmental change onto demographic rates and species interaction coefficients, which can be common across species and life stages or specific to each combination. While such an effort is feasible in simulations [101], the accurate empirical estimation of so many parameters in a natural system can require an impossible level of effort [5]. Integrating these parameters also increases model complexity and can complicate interpretation of modelling outcomes [102], in part because the likelihood of highly intercorrelated variables increases with an increasing number of parameters, thus raising the likelihood of spurious correlations [103] and issues with parameter identifiability [83]. Experimental manipulations of densities are ultimately required to tease apart whether positive or negative associations between species indicate interactions [104,105], but these can also be difficult to execute in systems larger than two–three species.

There are ways to reduce the dimensionality of parameterization for systems of multi-species interactions using discretization and grouping following general ecological rules and regularization approaches [106,107]. For one, species do not interact randomly but follow, in most cases, specific rules determined by species traits such as body size or height [3,37,108]. In addition, although interaction coefficients are usually expressed as *per capita* effects (e.g. [56]), the relative abundances of neighbouring species drive the frequency of interactions between individuals of different species, and with it, the overall biotic effects on the focal species [3]. In the absence of individual monitoring to quantify *per capita* effects, estimates based on trait-matching and abundance-based interaction frequency can be used as a first-principles baseline for grouping interactions in empirical systems and across life stages (figure 2). Furthermore, life-history information of the neighbours such as fast-growing versus slow-growing species or native versus invasive species can facilitate the interpretation of model coefficients as true interactions [109]. Causal inference methods also exist to better infer cause-effect relationships from observational data [110,111]. Lastly, relatively simple structured population models are able to capture a wide range of demographic variation within and among populations [112–114]. This means that the step from abundance-based data to demographic projections can potentially be achieved via relatively straightforward structured models, including as few as two stages (juveniles and reproductive).

We use the example of interactions among Mediterranean shrubs to highlight the minimum data required to do short-term forecasts (i.e. out-of-sample predictions) and climate-change projections (i.e. using scenarios of rainfall change) at the interface of population and community ecology. Using a dynamic N-mixture model [85] (figure 2), we showcase how ecological forecasts can be improved through more biologically realistic parameterizations of the effects of environmental conditions on population abundances, i.e. simultaneously projecting how biotic and abiotic factors affect the demography of interacting species.

## 5. Example: inferring demography from abundance datasets to project climate-change effects on interacting shrubs

**Figure 3.**
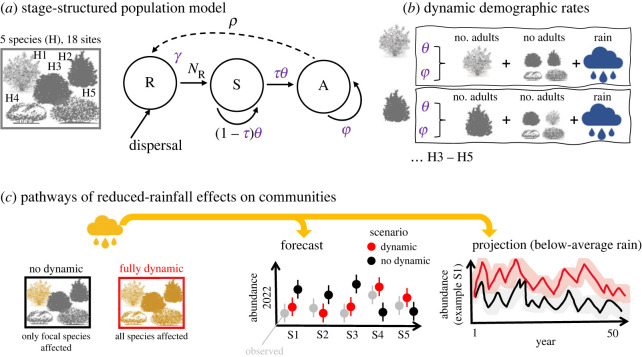
Overview of analyses for the Mediterranean shrubs showcasing applications of dynamic multi-species N-mixture models. (*a*) The local demography of five shrub species (H1–H5) across 18 plots is described by a three-stage life cycle (from which a matrix population model is built), in which transitions among seedlings (R), saplings (S) and reproductive adults (A) are defined by demographic rates: γ—gains of S, τ—transition S to A; θ,φ—survival of S and A, respectively, and ρ—*per capita* number of R produced by A. The demographic rate in purple (θ,φ,τ,γ) are inferred as latent states using a Bayesian hierarchical (N-mixture) model. The local demography is connected across the 18 sites by dispersal of R (via seeds produced by A). (*b*) For all species (here shown for two species only), θ,φ are modelled as functions of rainfall and intra- and interspecific adult (A) abundances. (*c*) By assuming changes in rainfall in the demographic rate models, either for all species simultaneously (fully dynamic) or for focal species only (no dynamic), the demographic pathways of climate change effects on the entire community can be assessed in near-term forecasts (out-of-sample predictions) and longer-term projections based on climate-change scenarios. Forecast skill can be assessed by comparing correlations between observed and predicted data. Note that plots are fictitious, showing a schematic overview of potential results.

Our case study focuses on understanding how feedbacks between demography and species interactions mediate changes in abundances of coexisting Mediterranean shrub species under increasingly drier winter weather (an overview of the analyses steps in the case study is presented in figure 3). We use data on the abundance of individuals of common shrub species in Doñana National Park (Spain) that have been recorded across 18 sites since 2007. Details on the study and sampling design are described in electronic supplementary material, S1. The original aim of the monitoring was to assess changes in community composition after a severe drought which led to a collapse of the shrubland in 2005 [115]. Recent evidence suggests that resilience to drought is well described by community-weighted species-specific demographic traits (e.g. longevity, reproductive output) [116], but dynamic forecasts of multi-species populations abundances have not been done thus far. Such forecasts are important because rainfall has become increasingly scarcer at the study site since the drought, a situation that is expected to continue under climate change [117,118]. However, how rainfall scarcity affects plant demography directly or indirectly via changing species interactions remains an open question, thus complicating assessments of the fate of the critical shrublands.

We used 8 years of data, collected across 18 plots between 2007 and 2021, on stage-specific abundances of the five most common shrub species in the study area (*Halimium commutatum, Halimium halimifolium, Rosmarinus officinalis, Lavandula stoechas* and *Cistus libanotis*) to estimate stage-specific survival (of saplings and reproductive adults), transitions of saplings to adults and gains of saplings to the population as latent, i.e. unobserved, states, inferred from the abundances (figure 3). We modelled these demographic rates jointly for all species using generalized linear models that included rainfall and inter- and intraspecific densities (adult abundances in 5 × 5 m plots) as covariates. Recruitment of seedlings (ratio seedlings*_t_*_+1_/adults*_t_*) was assumed to be fixed, as we only had two years of data on recruitment (see electronic supplementary material, S1). For each species, abundances at the beginning of time *t* were estimated from the demographic transitions from (*t* − 1) to *t.* Interactions among the shrubs are largely determined by size, i.e. the number of relatively large, adult neighbouring shrubs [119,120]. In the absence of information on the spatial location of the shrubs, we considered all adult shrubs in a 5 × 5 m plot as neighbours. We summed the abundance of adult interspecific neighbours at the beginning of time *t* and used this pooled measure of interspecific effects as covariate in the demographic rate models at *t* for each species (we included the abundance of intraspecific adults as a separate covariate). Electronic supplementary material, S1 showcases the Bayesian dynamic approach used to parameterize models, test their goodness of fit and test their ability to recover parameters from simulated data. This methodology can be readily applied to other community datasets [84] and to other drivers of global change, but our study also highlights that sufficient spatio-temporal replication of stage-specific counts is needed to avoid overparameterization and parameter unidentifiability. In our particular case, replication in space compensated for the relatively low temporal replication of 8 years, but spatial replication of similar studies is typically much larger [83,84]. The low spatio-temporal resolution of the data can increase uncertainties of parameter estimates (electronic supplementary material, figures S18 and S19) and, in our case, did not give us enough degrees of freedom to parameterize more complex and realistic covariate effects (e.g. interactions between rainfall and densities or quadratic rainfall effects).

We used predictions from the demographic rate models (electronic supplementary material, figures S2–S6) to build stage-structured metapopulation models for each species [120]. In these models, the local demography of every shrub species in each of the 18 plots was described by a stage-structured matrix population model (figure 3). The 18 local matrix models were joined by a dispersal matrix, which described adult plants producing seeds in a given plot that germinated as seedlings in adjacent plots (see electronic supplementary material, S1 for details). We validated how well the metapopulation model predicted observed abundances by projecting abundances of each species until 2021, starting with the site- and stage-specific population vector in 2010, and visually comparing the results to observed abundances. This *in-sample* validation showed a good fit to adult abundance data (observed abundances were within the 95% C.I. of predicted abundances for most species, sites and years; electronic supplementary material, figures S8–S13). Predicted abundances of saplings diverged more strongly from observed ones, likely because we estimated gains of saplings to the population as a constant and did not account for seed production when inferring demography from abundances. We simplified these processes as we lacked data for more complex models (electronic supplementary material, S1), highlighting that the lack of abundance data on several life cycle stages can be an important limiting factor on the detail of demographic inference from abundances.

**Figure 4.**
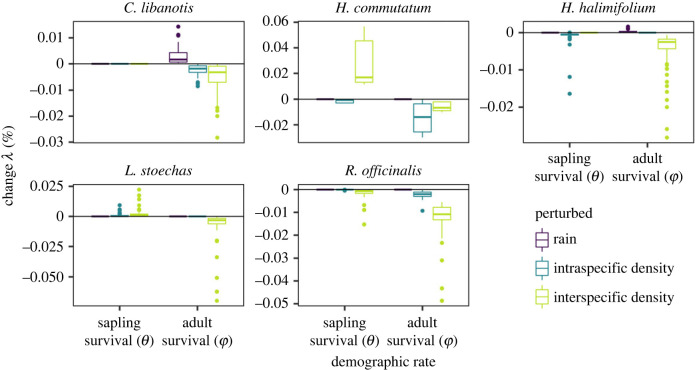
Percentage of change in population growth (*λ*), measured as the dominant eigenvalue of stage-structured matrix population models describing the local demography of five shrub populations, in response to increasing rainfall or intra- and interspecific densities by 10% when predicting sapling survival (*θ*) and adult survival (*φ*).

We then focused on local population dynamics of each shrub species and determined the relative sensitivity of the local population growth (*λ*) of each shrub species at equilibrium population density to changes in rainfall, intra- and interspecific densities. These sensitivity analyses demonstrate that population dynamics of most species are strongly influenced by negative effects of species interactions (including both intra- and interspecific densities) on adult survival—suggesting competition. Notably, however, for some species, such as *H. commutatum* and *L. stoechas*, such negative effects are balanced by positive effects of interspecific densities on juvenile survival (figure 4). As expected, rainfall positively affects populations, although the effect is not as strong as the effects of interspecific densities (figure 4). We performed additional sensitivity simulations in which we estimated the indirect effects of rainfall on population growth via changes in interspecific densities (see *Perturbation analyses* in electronic supplementary material, S1). These simulations show that the strength of such indirect effects differs among species and can be substantial, most importantly for *R. officinalis* where a 10% increase in rainfall affecting neighbours' survival decreases population growth by 8% on average (electronic supplementary material, figure S15). This system of interacting shrubs therefore suggests that changes in abiotic conditions can strongly affect the community via interactions among neighbouring species and contrasts with other systems of non-trophic interactions, where interspecific interactions affect demography far less than intraspecific interactions [36].

**Figure 5.**
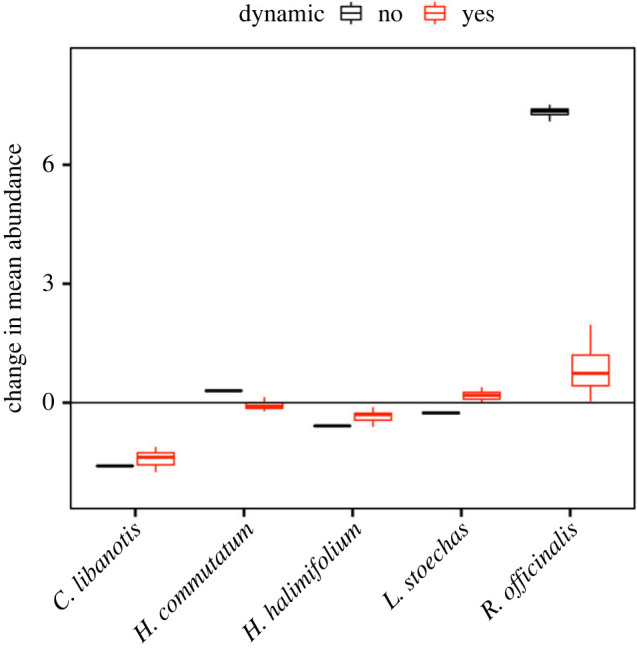
Projected changes in abundance estimates of interacting Mediterranean shrub species. Boxplots show distributions of changes in mean adult abundances (number of individuals in 5 × 5 m plots, averaged over 50 time steps and 100 simulation runs) across 18 sites (for more detailed plots including different stages and changes in variance see electronic supplementary material, figures S14 and S15). Changes are based on subtracting mean abundances under projections assuming climate change (sampling below-average rainfall with a probability of 0.8) from projections assuming past climate (randomly sampling past observed rainfall values). We either projected feedbacks between demography and species interactions (red) or sampled interspecific densities from past observed values without dynamically projecting them (black; electronic supplementary material, S1 for details).

Intra- and interspecific interactions mediate population responses of the shrubs when simulating a drier future. We jointly projected the metapopulations of the interacting shrubs using average posterior values of parameters (see [19,99] for examples of full uncertainty propagation in projections) and assuming a higher prevalence of below-average rainfall in the next 50 years (figure 3; electronic supplementary material, S1). Under these projections, not all species decrease in abundance compared to baseline projections (randomly sampling all rainfall values 2007–2021) (figure 5). Abundances of *C. libanotis* decrease most strongly, followed by *H. halimifolium*, as adult survival in both species is directly positively affected by rainfall. Plants may also experience more competition from shrubs not directly negatively affected by scarcer rainfall, as is the case for *H. commutatum*, *L. stoechas* and *R. officinalis*. Abundances of *H. commutatum* and *L. stoechas* do not change under drier conditions, as, in addition to not being directly affected by rainfall, a higher adult survival is countered by a lower juvenile survival under lower interspecific densities (figure 4). By contrast, abundances of *R. officinalis* increase under climate change (figure 5), as lower abundances of neighbours under climate change decrease the negative effect of interspecific density on adult and juvenile survival (figure 4).

**Figure 6.**
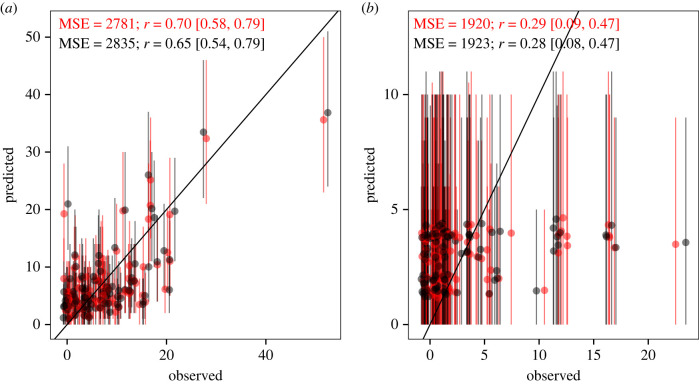
Comparison of observed and predicted abundances of adults (*a*) and saplings (*b*) in 2022. Points show abundances of all five modelled shrub species for each of 18 study plots (*n* = 90). Predicted abundances show averages ± 95% credible interval (error bars) from 2100 posterior samples of the Bayesian multi-species demographic model. The points and error bars on the *x*-axis are displaced slightly to facilitate visualization. We either predicted how rainfall changes affect all interacting species simultaneously (red; fully dynamic) or perturbed the effect of rainfall change on interspecific densities (black; no dynamic). Black lines show the 11 line indicating perfect fit between predicted and observed abundances. For each of the two forecast scenarios, mean squared error (MSE) and Pearson's correlation coefficients (*r*, mean [95% confidence interval]) were calculated on the average posterior abundance values to compare forecast skill.

Projections that do not account for climate-change effects on interspecific densities (no dynamics in figure 5) tend to show higher increases or decreases in abundances for most species compared with fully dynamic projections; they also increase the variation in abundance forecasts (electronic supplementary material, figure S17). Short-term forecasts of most-recently collected abundances (2022) lend further support to the importance of fully dynamic projections of abundance changes. Starting with stage-specific abundances in 2007, we forecast abundances to 2022, either assuming a fully dynamic model or a simplified one, i.e. assuming no climate effects on interspecific densities (see electronic supplementary material, S1 for details). We show that fully dynamic forecasts provide an accurate estimate of observed adult abundances in 2022 (but less so for saplings due to the data limitations in the relevant models, as discussed above; figure 6; electronic supplementary material, figure S14). Simplified assumptions in the forecasts, meanwhile, decrease forecast accuracy (figure 6). More years of independent data to evaluate the forecasts are needed for more conclusive results; this is particularly true for mechanistic forecasts that include many parameter and lagged effects of density (figure 1; [8,9]). Nevertheless, several lines of evidence in our case study suggest that omitting a crucial pathway of climate change on populations in the forecast, i.e. interspecific interactions affecting stage-specific survival, can lead to an inaccurate interpretation of population dynamics under climate change.

Winters in the Mediterranean have been getting drier over the past 10 years; projections of our dynamic multi-species demographic models are in line with empirical evidence of abundance trends (electronic supplementary material, S1) and literature that shows a higher recent mortality of *H. halimifolium* and increases in abundances in *L. stoechas* [115]. Based on life-history theory, we would expect populations of relatively short-lived species such as *L. stoechas* [116] to be more sensitive to changes in abiotic conditions than populations of longer-lived species such as *H. halimifolium* [122]. However, indirect effects of such changes via species interactions can have substantial effects on natural communities across different life histories [28,60]. The models we developed for the shrubs are relatively simple and likely do not incorporate all relevant biological processes (all scripts and data can be found at: https://github.com/MariaPaniw/shrub_forecast; [123]). We will update our models iteratively [124] as we gain a better understanding of size-mediated demographic rates, spatially explicit interactions and hidden demographic stages, such as seed banks [125]. However, these dynamic models highlight the importance of a better integration of the pathways through which environmental change can affect communities. Perturbing these pathways creates different forecasting scenarios and can result in more nuanced decision making on the management of this community where the focus can vary between managing populations (e.g. of the cover of the most common species, *H. halimifolium*) and communities (e.g. introducing burning to entire patches to promote seedling recruitment and thus higher food availability for rodents) [126].

## 6. Conclusion and future directions

Assessing individual, age- or stage-specific demographic responses to biotic and abiotic drivers for several species in a community simultaneously offers an essential perspective into the fates of populations and communities under environmental change. Projections of population dynamics based on abundance data have been demonstrated to be only as accurate as projections based on demographic models in some systems [127]. However, as our case study shows, this may not be the case for the many communities where even small environmental changes can substantially change community dynamics by altering the outcomes of species interactions [128]. Similarly, averaging these direct and indirect effects of environmental change over demographic stages and even populations can substantially obscure our ability to assess the capacity of communities to bounce back from perturbations [83,129]. This occurs when different demographic rates respond distinctly to intra- and interspecific densities, so that decreases in one life cycle stage may be compensated by increases in another. Such context-dependent demographic responses are likely very common in nature [62,130] and challenge classic assumptions of what taxa are most sensitive to global change [131]. Robustly capturing some of the pathways through which global change affects populations may allow us to design robust alternative scenarios of outcomes under global change, thus avoiding the ‘forecast trap’ (*sensu* [132,133]) where management decisions rely too closely on optimizing the forecast ability of a single best-fit model.

Our case study demonstrates that advances in statistical tools [83] and data integration [134], including life-history information, known effects of heterospecific and conspecific neighbours, or dispersal, make it possible to explore demography–biotic interaction feedbacks in a wide range of systems. However, more studies aimed at improving the parameterization and biological realism of multi-species demographic inference are needed. The methods presented here require that sufficient count data be available to infer demographic rates. This may preclude the inclusion of less abundant and potentially more threatened species. Such challenges can be partially overcome by the inclusion of informative priors into Bayesian model fitting [89], and studies that integrate informative priors into multi-species demographic inference are needed. In addition, populations of many species are structured by continuous traits, such as size or body mass, and many community-level studies record not only counts but also such key traits. Future studies that parameterize demographic rates as functions of continuous traits, in turn affected by species interactions, may not only address an important biological pathway of global-change effects on populations and communities [84,135], but can also result in more efficient model fitting that requires fewer parameters [136].

## Data Availability

All data to replicate the analyses in this manuscript have been deposited on Dryad: https://doi.org/10.5061/dryad.8cz8w9gvc [137]. R scripts to process the data and run all analyses presented in the manuscript are available at: https://github.com/MariaPaniw/shrub_forecast [123]. The data are provided in the electronic supplementary material [138].
